# Melflufen, a peptide‐conjugated alkylator, is an efficient anti‐neo‐plastic drug in breast cancer cell lines

**DOI:** 10.1002/cam4.3300

**Published:** 2020-07-27

**Authors:** Alexander Schepsky, Gunnhildur Asta Traustadottir, Jon Petur Joelsson, Sævar Ingthorsson, Jennifer Kricker, Jon Thor Bergthorsson, Arni Asbjarnarson, Thorkell Gudjonsson, Nina Nupponen, Ana Slipicevic, Fredrik Lehmann, Thorarinn Gudjonsson

**Affiliations:** ^1^ Stem Cell Research Unit Biomedical Center University of Iceland Reykjavik Iceland; ^2^ Department of Laboratory Hematology University Hospital Landspitali Reykjavik Iceland; ^3^ Cancer Research Laboratory Biomedical Center Reykjavik Iceland; ^4^ Oncopeptides Stockholm Sweden

**Keywords:** alkylator, aminopeptidases, breast cancer, breast cancer cell lines, Melflufen

## Abstract

Melphalan flufenamide (hereinafter referred to as “melflufen”) is a peptide‐conjugated drug currently in phase 3 trials for the treatment of relapsed or refractory multiple myeloma. Due to its lipophilic nature, it readily enters cells, where it is converted to the known alkylator melphalan leading to enrichment of hydrophilic alkylator payloads. Here, we have analysed in vitro and in vivo the efficacy of melflufen on normal and cancerous breast epithelial lines. D492 is a normal‐derived nontumorigenic epithelial progenitor cell line whereas D492HER2 is a tumorigenic version of D492, overexpressing the HER2 oncogene. In addition we used triple negative breast cancer cell line MDA‐MB231. The tumorigenic D492HER2 and MDA‐MB231 cells were more sensitive than normal‐derived D492 cells when treated with melflufen. Compared to the commonly used anti‐cancer drug doxorubicin, melflufen was significantly more effective in reducing cell viability in vitro while it showed comparable effects in vivo. However, melflufen was more efficient in inhibiting metastasis of MDA‐MB231 cells. Melflufen induced DNA damage was confirmed by the expression of the DNA damage proteins ƴH2Ax and 53BP1. The effect of melflufen on D492HER2 was attenuated if cells were pretreated with the aminopeptidase inhibitor bestatin, which is consistent with previous reports demonstrating the importance of aminopeptidase CD13 in facilitating melflufen cleavage. Moreover, analysis of CD13^high^ and CD13^low^ subpopulations of D492HER2 cells and knockdown of CD13 showed that melflufen efficacy is mediated at least in part by CD13. Knockdown of LAP3 and DPP7 aminopeptidases led to similar efficacy reduction, suggesting that also other aminopeptidases may facilitate melflufen conversion. In summary, we have shown that melflufen is a highly efficient anti‐neoplastic agent in breast cancer cell lines and its efficacy is facilitated by aminopeptidases.

## Graphical abstract

Melflufen is a lipophilic peptide‐conjugated alkylator that is converted by aminopeptidases inside cells to hydrophilic melphalan metabolites. Melflufen is currently in clinical trials in late stage myeloma where it has shown promising effects. However, studies on the effect of melflufen on solid tumors are limited. Here, we have analyzed the efficacy of melflufen in isogenic normal and cancerous breast epithelial lines as well as the triple‐negative MDA‐MB‐231 in 2D, 3D and in vivo. Our data shows that Melflufen is more potent then Melphalan or Doxorubicin and its efficacy is facilitated by aminopeptidases.

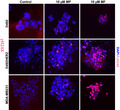

## 1 INTRODUCTION

Breast cancer is a heterogeneous group of diseases divided into several subclasses with distinct prognosis and survival outcomes. Even though the 5‐year survival rate of breast cancer is over 90%, due to the high prevalence, it is a leading cause of cancer‐related death in women.^1^, ^2^ Based on gene expression analysis, breast cancer can be classified into five subclasses, Normal‐like, Luminal A and B, HER2 amplified, and triple negative (TN) with TN and HER2 amplified breast cancer patients having the worst prognosis.^1^, ^3^, ^4^

Cellular origin of breast cancer is debated, but many studies link it to resident stem or progenitor cells in the breast gland.^5^, ^6^ Modeling breast morphogenesis and cancer progression in vitro using cells with stem cell properties is important to both unravel the cellular and molecular mechanism of breast cancer and to improve screening for efficient cancer drugs.^2^, ^7^

D492 is a breast epithelial cell line generated from reduction mammoplasty.^2^, ^8^ Its stem cell‐like properties are evident in its ability to propagate to both luminal and myoepithelial cells in culture. In the 3D microenvironment, D492 generates branching structures similar to terminal duct lobular units (TDLUs) in vivo.^8^ D492HER2 is a cancerous derivative of the D492 line generated by overexpression of the HER2 oncogene.^9^ In contrast with the D492 line, which is nontumorigenic, D492HER2 forms aggressive tumors in mice.^9^ These isogenic cell lines have been used to study the molecular mechanism of branching morphogenesis and cancer progression in the breast gland.^2^, ^9^, ^10^, ^11^, ^12^

Melphalan is a well‐known cancer drug used for decades for the treatment of various cancer types, and it is still widely used for hematological cancers today.^13^ It is an alkylating agent that works by adding an alkyl group to the guanine base of the DNA, resulting in an aberrant linkage between DNA strands, DNA breakage, and inhibition of DNA synthesis.^14^ Melphalan is a hydrophilic drug, and as such, it does not penetrate cell membranes easily, which is limiting to its anticancer potential. In contrast, melflufen (melphalan flufenamide), a new peptide‐conjugated alkylator, is highly lipophilic and therefore penetrates the cell membrane easily.^15^ Inside the cell, melflufen is rapidly hydrolyzed into less lipophilic metabolites that retain high alkylating potential, leading to their entrapment and accumulation.^15^

Aminopeptidases belong to a large family of enzymes that catalyze the cleavage of amino acids from the amino terminus of proteins or peptides and are shown to facilitate intracellular hydrolysis of melflufen.^16^ Aminopeptidases are involved in multiple cellular processes, and their expression and activity are frequently deregulated in cancer cells.^16^ For this reason, aminopeptidase inhibitors bestatin and tosedostat have been investigated in the clinical settings.^16^ Exploiting aminopeptidase activity to convert a lipophilic drug such as melflufen to an intracellular hydrophilic metabolite with high alkylating potential may offer an effective alternative therapeutic approach. Previously it has been shown that aminopeptidase N (ANPEP or CD13) can hydrolyze melflufen, but it is largely unknown whether melflufen can be a substrate for other aminopeptidases.

Application of melflufen has mainly been focused on multiple myeloma, where it has shown promising results in phase 3 clinical trial in relapse and refractory patient population.^13^ Yet it is unexplored whether melflufen can be effective in other cancer types such as breast cancer. Here, we demonstrate the efficacy of melflufen in D492HER2 breast epithelial cells overexpressing the HER2 oncogene and the triple‐negative cell line MDA‐MB‐231.

## 2 MATERIAL AND METHODS

### 2.1 Cell culture

D492 and D492HER2 cells were cultured in H14 media supplemented with penicillin (100 U/mL) and streptomycin (100 µg/mL) in culture flasks coated with collagen I (Advanced Biomatrix), as described previously.^1^ MDA‐MB‐231 cells (ATCC^®^ HTB‐26^™^) were maintained in Gibco™ DMEM media containing 10% fetal bovine serum (FBS) (Invitrogen) supplemented with penicillin (100 U/mL) and streptomycin (100 µg/mL). All cells were maintained at 37°C and 5% CO_2_ and subcultured at a ratio of 1:10, 1‐2 times/week. For 3D culture, 8000 cells were seeded on top‐of Matrigel (Corning) and cultured for 5‐days in H14 or DMEM medium, changing media every 2 days. After colonies were formed, drugs were added to the media and the effects on 3D colonies were visualized with anEVOS FL Auto 2 Cell Imaging System (Thermo Fisher Scientific).

### 2.2 Drugs

Melflufen and melphalan were obtained from Recipharm, Sweden, dissolved in DMSO and stored at −80°C. Doxorubicin was obtained from Actavis, Iceland. Bestatin was purchased from Sigma Aldrich and resuspended according to the manufacturer´s protocol. All substances were further diluted in cell culture media to appropriate concentrations prior to the start of experiments.

### 2.3 Proliferation and cell viability assays

For proliferation assays 10 000 cells were seeded in 96 well plates, cultured for 24 h prior to the addition of drugs and inhibitors. Cells were incubated with various drug concentrations in medium for 30 min thereafter medium was refreshed. Proliferation was monitored using an IncuCyte ZOOM (Sartorius). Graphical analysis of cell numbers was performed for selected time points, 48 and 72 h post drug application. Cell viability was assessed in a similar way but using a colorimetric assay with PrestoBlue^TM^ cell viability reagent (Invitrogen).

### 2.4 Protein isolation and Western blotting

Total protein content was extracted from cells using RIPA buffer (50 mmol L^‐1^ tris‐HCl (pH 7.4), 150 mmol L^‐1^ NaCl, 0.5% Igepal, 5 mmol L^‐1^ EDTA (pH 8.0), 0.1% SDS supplemented with Halt Protease, and Phosphatase Inhibitor cocktail (Thermo Fisher). Equal volume of each sample was resolved on Tris‐glycine SDS‐polyacrylamide gels and transferred to a nitrocellulose membrane (Santa Cruz Biotechnology), blocked in blocking buffer (5% skim milk powder in PBS), and probed with primary antibodys overnight at 4°C (Actin B, Li‐Cor Cat. No. 926‐42212; γH2AX, Cell Signaling Technology, Cat. No. 80 312; H2AX, R&D Systems, Cat. No. MAB3406; DPP7, Proteintech, Cat. No. 19018‐1‐AP; LAP3, Santa Cruz Biotechnology, Cat. No. sc‐398601; CD13, Santa Cruz Biotechnology, Cat. No. sc‐51522; cleaved PARP (Asp214), Cell Signaling Technology, Cat. No. 5625; cleaved Caspase‐3 (Asp175), Cell Signaling Technology, Cat. No. 9664). Proteins were detected using horseradish peroxidase‐conjugated secondary antibodies (Santa Cruz Biotechnology) and Pierce ECL detection substrate (Thermo Fisher). All western blot assays were replicated in triplicate, resolved bands normalized against actin and quantified using FIJI.

### 2.5 Gene expression

Total RNA was extracted with TRI‐Reagent (Life Technologies) and reverse transcribed using the SuperScript® IV First‐Strand Synthesis System (Invitrogen). Quantitative real‐time PCR was carried out using Luna^®^ Universal qPCR Master Mix (New England Biolabs) with predesigned primer pairs CD13 (Hs.PT.56a.534906), LAP3 (Hs.PT.58.2647348), DPP7 (Hs.PT.58.39153978), using ß‐2‐microglobulin (Hs.PT.58v.18759587) or GAPDH (Hs.PT.39a.22214836) as reference. Comparative C_T_ values were determined in triplicate using an ABI 7500 Real Time PCR instrument (Applied Biosystems).

### 2.6 Transient knockdown of aminopeptidases by siRNA

Pre‐designed Silencer® Select siRNAs against CD13 (s683), LAP3 (s27311) and Silencer® Select Negative Control No. 1 (ThermoFischer Scientific), as well as siRNA against DPP7 (Santa Cruz Biotechnology) were used at a concentration of 10 nmol L^‐1^. For delivery of siRNA, Lipofectamine® RNAiMAX tranfection reagent (ThermoFischer Scientific) was used according to the manufacturer´s protocol. 48 h post‐transfection cells were incubated with the indicated drug concentrations for 30 min, and then cell viability was monitored using an IncuCyte ZOOM (Sartorius) or using a colorimetric assay with PrestoBlue^TM^ cell viability reagent (Invitrogen) 48 h post‐incubation. Successful knockdown of target genes at the time point of drug application was confirmed by qRT‐PCR and Western blot.

### 2.7 Immunostaining and Confocal imaging

Immunofluorescence was captured and visualized using an Olympus FV1200 confocal microscope (Olympus, Tokyo, Japan). Cells were fixed in formalin for 20 mins before staining. Antibodies used were BV421 γH2AX (pS139) (BD Horizon, Cat. No. 564 720), 53BD1 (R&D Systems, Cat. No. MAB18772), yH2Ax (pS139) (R&D Systems, Cat. No. AF2288), Alexa‐Fluor phalloidin (Life Technologies, Cat. No. A12379), was used to stain for actin. DAPI was used for nuclear staining.

### 2.8 Chick embryo chorioallantoic grafting assay

Fertilized White Leghorn eggs were incubated at 37.5°C with 50% relative humidity for 9 days (Inovotion INC, France). On day E9, the chorioallantoic membrane (CAM) was dropped down by drilling a small hole through the eggshell into the air sac, and a 1 cm^2^ window was cut in the eggshell above the CAM. MDA ‐MB‐231 or D492HER2 cells were detached with trypsin, washed with complete medium and suspended in graft medium. An inoculum of 1 × 10^6^ cells was added onto the CAM of each egg. Eggs were then randomized into eight groups. On day E10, tumors became detectable, and were treated with either vehicle (1% DMSO in 1x PBS), doxorubicin at 50 µmol L^‐1^ or melflufen at 12 µmol L^‐1^, 50 µmol L^‐1^, and 200 µmol L^‐1^ per egg. For all conditions, the injection volume of 100 μl/egg, was dropped onto the tumor. On day E18 the upper portion of the CAM containing tumor was removed, washed in PBS and then directly transferred in PFA (fixation for 48 h). The tumor was then washed, carefully cut away from normal CAM tissue and weighed. Analysis of metastasis was done in parallel. Briefly, a 1 cm^2^ portion of the lower CAM was collected to evaluate the number of metastatic cells. Genomic DNA was extracted from the CAM and analyzed by qPCR with specific primers for human Alu sequences. Calculation of Cq for each sample, mean Cq and relative amount of metastasis for each group was performed with the Bio‐Rad® CFX Maestro® software. To estimate toxicity, eggs were checked at least every two days, for viability and visible macroscopic abnormalities. The number of dead embryos counted on day E18, combined with reported abnormalities was used to evaluate total toxicity.

### 2.9 Statistical analysis

Two‐way analysis of variance (ANOVA) using Tukey's multiple comparison test was performed using GraphPad Prism to test significance. *P*‐values below 0.05 were considered significant (*P ≤ .05, **P ≤ .01, ***P ≤ .001, ****P ≤ .0001).

## 3 RESULTS

### 3.1 Breast cancer cells are more sensitive to melflufen than normal cells

D492 is a breast epithelial progenitor cell line that can generate luminal and myoepithelial cells in monolayer culture while in 3D culture it forms elaborate branching structures akin to terminal duct lobular units (TDLUs) in the breast^2^, ^3^, ^4^ (Figure S1, left). D492HER2 is an oncogenic derivative of D492 generated by overexpressing the ErbB2(HER2) oncogene and in 3D culture it forms grape‐ and spindle‐like colonies^5^ (Figure S1, right). Due to their isogenic nature and serum free cell culture condition, D492 and D492HER2 are ideal for drug screening.

**FIGURE 1:**
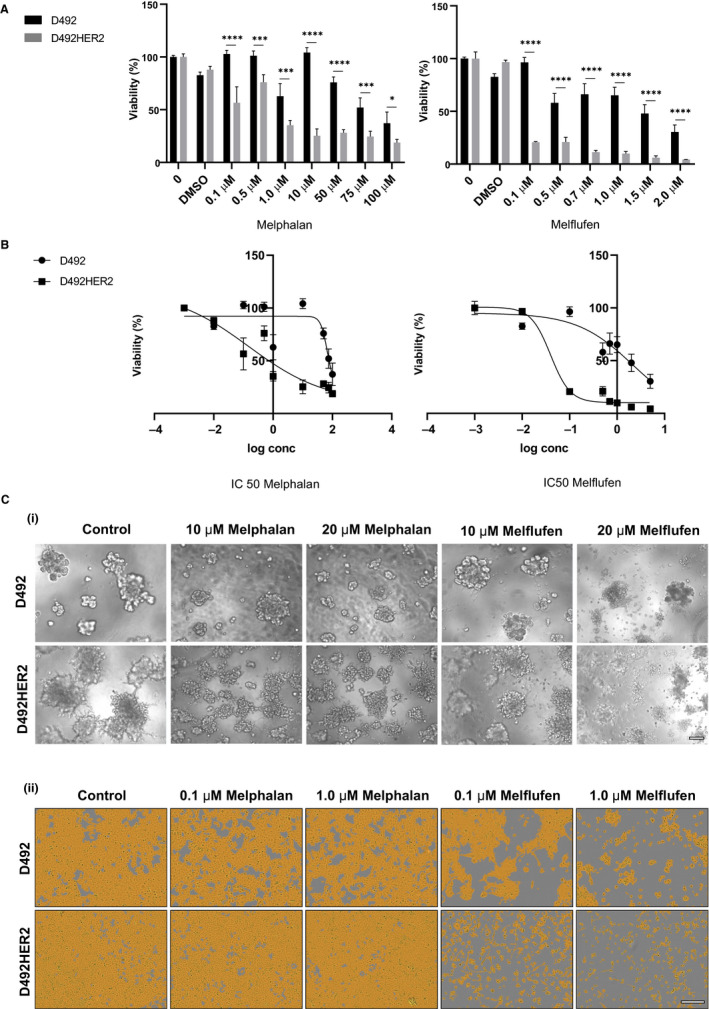
Melphalan and melflufen reduce cell viability of cancerous breast epithelial cells more efficiently than isogenic normal epithelial cells. (A) D492 and D492HER2 cells were seeded in 96 well plates and treated with various concentrations of either melphalan or melflufen and cell viability was assessed 48 h post‐treatment. Shown are means ± standard deviation ( n = 3). (B) From the mean of three independent experiments, IC50 values were calculated. Concentrations are presented in log scale on x axes while y axes represent cell viability. n = 3. (C) D492 and D492HER2 cells were cultured on top of reconstituted basement membrane (rBM) to generate branching and grape/spindle‐like structures respectively. After colony formation, cells were incubated with indicated concentrations of either melphalan or melflufen and phenotypic changes monitored. Scalebar = 100 μm

Here, we compared the efficacy of melphalan and melflufen in 2D and 3D culture in D492 and D492HER2 cell lines. D492 and D492HER2 cells were treated with different doses of melphalan and melflufen. Melphalan was added in concentrations ranging from 0.1 µmol L^‐1^ up to 100 µmol L^‐1^ while, concentrations for melflufen were in the range from 0.1 µmol L^‐1^ to 2 µmol L^‐1^ (Figure 1A).

D492HER2 cells showed increased sensitivity to melphalan compared to the progenitor cell line D492 (Figure 1A, left). However, concentration as high as 100 µmol L^‐1^ was insufficient to kill the whole cell population. In both cell lines, around 40% of D492 cells and at least 20% of D492HER2 cells were still viable after incubation with 100 µmol L^‐1^ melphalan. On the contrary, melflufen decreased viability of both D492 and D492HER2 cells at much lower doses (Figure 1A, right). While around 60% of the D492 cells were still viable at 1 µmol L^‐1^ dose of melflufen, D492HER2 cells were reduced to less than 10%. Importantly, viability of D492HER2 cells was significantly more affected by treatment with melflufen compared to D492 for all doses investigated. The IC50 numbers, 48h post drug application, show that D492HER2 cells were nearly 10 times more sensitive to melflufen than D492 cells (Figure 1B). Collectively, this demonstrates that melflufen is more potently affecting the cancerous D492HER2 cell line than its normal‐derived isogenic cell line D492.

It is well‐known that gene expression in cells is highly dependent on the external environment such as composition of cell culture media and the surrounding microenvironment.^6^ Thus, whether cells are cultured in monolayer (2D) or in 3D extracellular matrix can greatly affect their gene expression. It is, therefore, likely that cells respond differently to drugs when cultured in 2D vs 3D environment. To address this, we seeded D492 and D492HER2 cells on top of reconstituted basement membrane (rBM, Matrigel). In this assay, cells can generate colonies that capture their phenotypic traits better than in 2D culture. In 3D environment, D492 cells form TDLU like structures whereas D49HER2 cells form grape‐like and/or mesenchymal like colonies (Figure 1C, i and Figure S1). When 3D cultures of D492 and D492HER2 cells were treated with various doses of melphalan and melflufen, the cultures lost their morphogenetic features in a dose dependent manner (Figure 1C, i). Interestingly, while in 2D culture, both cell lines showed severe signs of apoptosis already with 1 μmol L^‐1^ melflufen (Figure 1C, ii), concentrations needed to be raised to 10 μmol L^‐1^ and 20 μmol L^‐1^ for D492HER2 and D492 cells, respectively, in order to see any effect on colony growth and morphogenesis in 3D culture (Figure 1C, i)). Again, carcinogenic D492HER2 cells were more sensitive to melflufen exposure than D492 cells. Similar concentrations of melphalan did not affect the cells in same manner (Figure 1C, i).

### 3.2 Melflufen activity is modulated by aminopetidases

**FIGURE 2:**
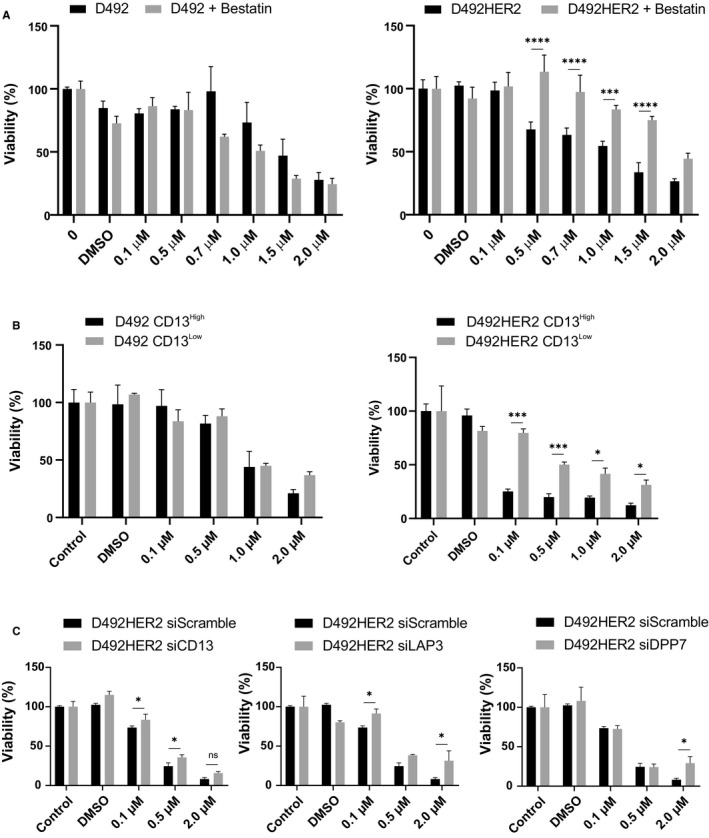
Melflufen activity is modulated by aminopeptidases. (A) D492 and D492HER2 cells were pretreated with the aminopeptidase inhibitor bestatin (10 µmol L^‐1^) for 1 h prior to incubation with increasing concentrations of melflufen and cell viability analysed 48 h later. Shown are means ± standard deviation (n = 3). (B) CD13 high and low FACS sorted D492 and D492HER2 cell populations were incubated with indicated amounts of melflufen for 2 h. Cell proliferation was analysed via an Incucyte and cell viability calculated after 48 h. Shown are means ± standard deviation (n = 3). (C) D492HER2 cells were siRNA depleted for specific aminopeptidases as indicated. 24 h post depletion, cells were incubated with indicated concentrations of melflufen and cell viability analysed via Incucyte. Shown are means ± standard deviation (n = 3)

As the lipophilic drug melflufen readily penetrates cell membranes, its cytotoxic effect is largely based on accumulation of its more hydrophilic metabolites within cells. Aminopeptidases are thought to be responsible for the cleavage of melflufen, and indeed, in our model, the aminopeptidase inhibitor bestatin attenuated the activity of melflufen in D492HER2 cells (Figure 2A, right). Interestingly, in D492 cells, preincubation with bestatin showed no effect on melflufen activity (Figure 2A, left).

CD13 is the most studied member of the aminopeptidase family and evaluation of endogenous CD13 expression levels revealed that D492HER2 cells have a 1.6‐fold increased expression level (Figure S2) when compared to D492 cells. In order to elucidate the importance of CD13 on sensitivity of D492 and D492HER2 cells to melflufen, we sorted D492 and D492HER2 cells into CD13^high^ and CD13^low^ expressing cell populations and subsequently treated these cells with melflufen. Interestingly, while no significant difference could be seen in neither CD13^high^ or CD13^low^ populations in D492 cells, the D492HER2 CD13^high^ cell population was more sensitive to melflufen than D492HER2 CD13^low^ cells (Figure 2B). This strongly indicates that high expression of CD13 is important for increased efficacy of melflufen in this tumorigenic cell line.

Although CD13 has been in the spotlight as the aminopeptidase responsible for the action of melflufen, the family of aminopeptidases is large, and it is possible that other aminopeptidases play a role as well. Here, we used siRNA to knock down three aminopeptidases, CD13, LAP3, and DPP7 in D492 and D492HER2 cells, and evaluated whether this affected the sensitivity of the cells toward melflufen. Successful knockdown of target aminopeptidases was confirmed by western blot (Figure S3). Indeed, with knockdown of either CD13 or LAP3, D492HER2 cells become less sensitive toward melflufen. On the contrary, knockdown of DPP7 had little effect except when cells were treated with high doses (2 µmol L^‐1^) of melflufen (Figure 2C). Collectively, this demonstrates that the activity of melflufen is highly dependent on the presence and activity of aminopeptidases and that CD13, as well as other aminopeptidases are involved.

### 3.3 Melflufen is more potent than doxorubicin in treatment of D492HER2 and MDA‐MB‐231 in vitro

**FIGURE 3:**
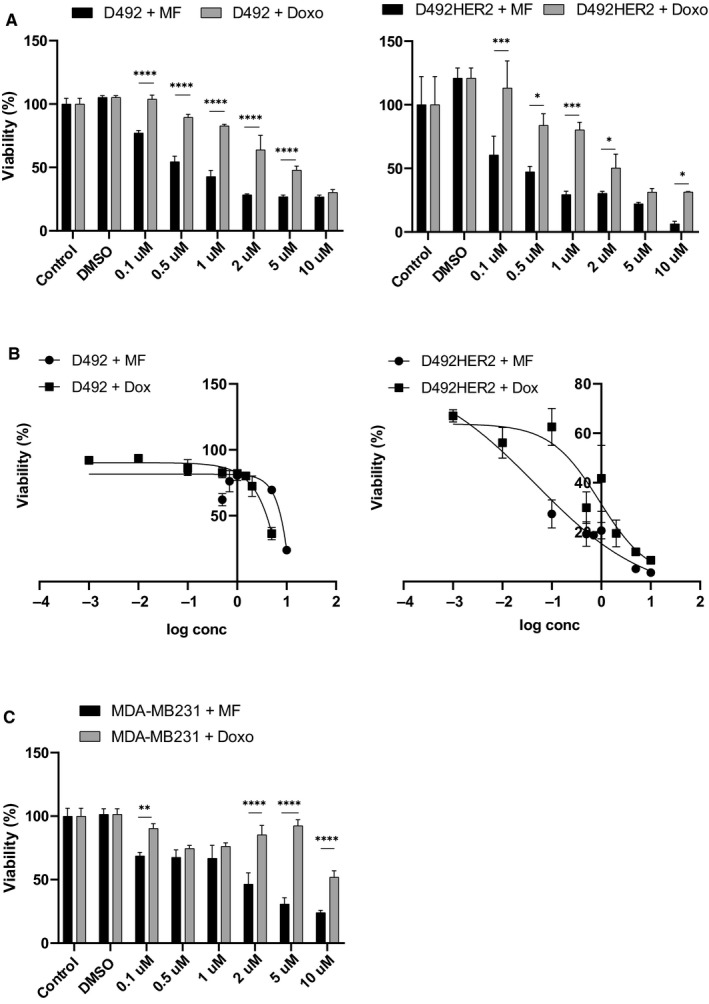
Melflufen reduces cell viability to a larger extent than doxorubicin. (A) D492 and D492HER2 cells were incubated with indicated concentrations of either melflufen or doxorubicin for 30 min. 48 h post‐treatment, cell viability was analysed via Prestoblue staining. Shown are means ± standard deviation (n = 3). (B) From the mean of three independent experiments, IC50 values were calculated. Concentrations are presented in log scale on x axes while y axes represent cell viability. (C) Triple‐negative cell line MDA‐MB231 was incubated with indicated concentrations of melflufen or doxorubicin for 30 min and cell viability analysed via Prestoblue staining 48 h post‐treatment. Shown are means ± standard deviation (n = 3)

Doxorubicin is a commonly used chemotherapeutic agent in the treatment of breast cancer. Therefore, we were intrigued to compare the efficacy of melflufen and doxorubicin on cell viability in our breast cell lines. Indeed, we showed that both D492 and D492HER2 cells are more sensitive to melflufen than doxorubicin treatment. While doses of up to 2 µmol L^‐1^ melflufen decreased cell viability dramatically in both cell lines, a dose in the range of 5 µmol L^‐1^ to 10 µmol L^‐1^ of doxorubicin caused a decrease in viability of these cells (Figure 3A). IC50 values for melflufen of 0.046 µmol L^‐1^ in D492HER2 cells and 0.52 µmol L^‐1^ in D492 cells compared to doxorubicin values of 0.92 µmol L^‐1^ for D492HER2 cells and 1.8 µmol L^‐1^ for D492 underpin these findings (Figure 3B). Having established this, we were also keen to test the effect of melflufen on the commonly used triple‐negative breast cancer cell line MDA‐MB231. While these cells tolerated higher drug concentrations for both drugs compared to our D492 cell lines, melflufen was significantly more efficient than doxorubicin in killing the MDA‐MB‐231 cells (Figure 3C).

### 3.4 Melflufen induces DNA damage in sensitive cells

**FIGURE 4:**
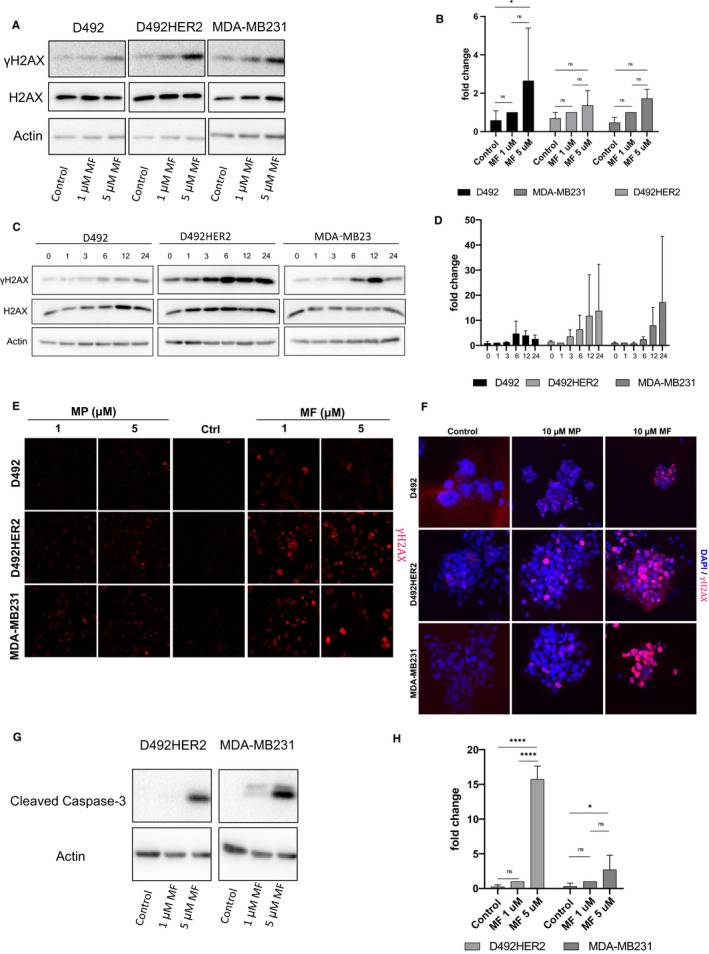
Meflufen induces DNA damage and apoptosis in cancer cells. (A) D492, D492HER2, and MDA‐MB231 cells were seeded on a six well plate and incubated with either none, 1 µmol L^‐1^ or 5 µmol L^‐1^ melflufen for 30 min and harvested 3 h post‐treatment. Equally loaded protein lysates subjected for immunoblotting for H2AX/ yH2Ax One representative blot of three is shown. (B) Quantification of protein bands of three independent experiments performed in 4A. Actin was used as loading control. Quantification was perfomed by FIJI. Shown are means ± standard deviation (n = 3). (C) D492, D492HER2, and MDA‐MB231 cells were incubated with 1 µmol L^‐1^ of melflufen for 30 min and harvested on indicated timepoints and subjected for immunoblotting for H2AX/ yH2Ax. One representative blot of 3 is shown. (D) Quantification of protein bands of three independent experiments performed in 4C. Actin was used as loading control. Quantification was perfomed by FIJI. Shown are means ± standard deviation (n = 3). (E) D492, D492HER2, and MDA‐MB231 cells were cultured in monolayer and 24 h after treatment with none, 1 µmol L^‐1^ or 5 µmol L^‐1^ melflufen or melphalan, cells were fixed and stained for yH2Ax (red). IF analysis reveals that melflufen increases DNA damage in a dose dependent manner. Melphalan shows only a minor increase in staining at the concentrations used. Scalebar = 30 μm. (F) For immunostaining of D3 cultures, colonies of D492, D492HER2 and MDA‐MB231 were incubated with either 10 µmol L^‐1^ melflfufen or melphalan for 2h, fixed 72h post‐treatment and stained for yH2Ax (red) and DAPI (blue). Scalebar = 100 µm (G) D492HER2 and MDA‐MB231 cells were incubated with increasing amounts of melflufen for 24 h, harvested in RIPA buffer and subjected to immunoblotting for cleaved caspase‐3. One representative blot of 3 is shown. (H) Quantification of protein bands of 3 independent experiments performed in 4G for caspase‐3. Actin was used as loading control. Quantification was perfomed by FIJI. Shown are means ± standard deviation (n = 3)

In order to elucidate in more detail, the mechanism of action of melflufen we analysed the amount of DNA damage in all three different cell lines after treatment with melflufen. In particular, we looked at the phosphorylation status of the protein H2AX and accumulation patterns of 53BP1, two proteins which have previously been shown to be involved in DNA damage.^7^ We observed a dose‐dependent increase in the phosphorylation of H2AX (ƴH2AX) in all three cell lines upon treatment with melflufen(Figure 4A and B, additional western blots used for statistical evaluation are shown in Figure S5A). Furthermore, time course experiments using a 1 µmol L^‐1^ single dose of melflufen revealed that melflufen was able to induce stable DNA damage for at least 24 h after treatment (Figure 4C) which increased over time (Figure 4D,, additional western blots used for statistical evaluation are shown in Figure S5B). To analyse these findings in more detail we performed immunofluorescence staining of cells treated with melflufen. When treated with melflufen in 2D culture, both carcinogenic cell lines, D492HER2 and MDA‐MB231 showed increased levels of ƴH2AX (Figure 4E), whereas D492 cells were minimally affected. Similar doses of melphalan only resulted in minor increase in ƴH2AX compared to melflufen. In addition, in 3D culture, a 10 µmol L^‐1^ dose of melflufen resulted in increased ƴH2AX staining (Figure 4F) as well as increased levels of 53BP1 (Figure S4) in contrast to melphalan that did not lead to such drastic increases at comparable doses. Again, both tumorigenic cell lines showed higher levels of DNA damage than the D492 cell line. Furthermore, increasing amounts of melflufen lead to cellular apoptosis in a dose dependent manner as revealed by Western blot for cleaved caspase‐3 (Figure 4G and H, additional western blots used for statistical evaluation are shown in Figure S5C) and cleaved PARP(Figure S5D) 24 h post‐treatment.

### 3.5 Melflufen is superior to doxorubicin in reducing the metastatic potential of MDA‐MB‐231 cells in vivo

**FIGURE 5:**
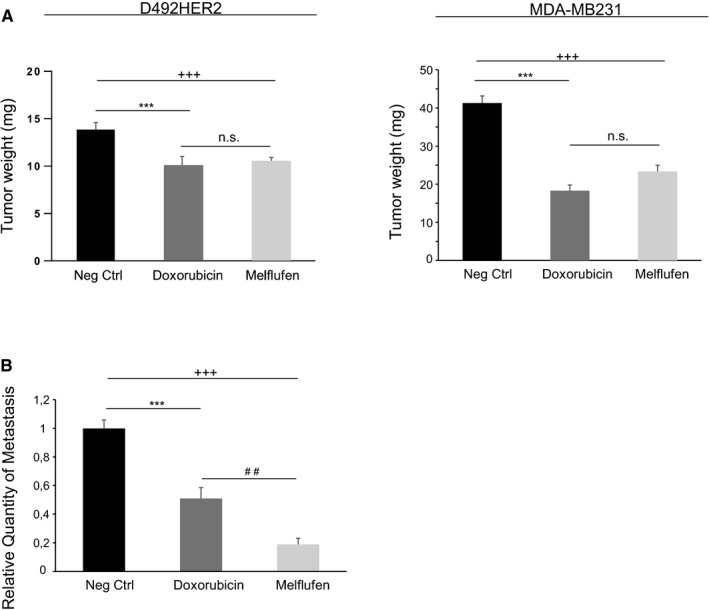
In vivo effects of Melflufen on MDA‐MB‐231 cells. (A) D492HER2 and MDA‐MB231 cells were xenograted on chicken chorioallantoic membranes (CAM) and formed tumors were treated with 50 µmol L^‐1^ melfufen or doxorubicin and analyzed by weight. Shown are means ± standard deviation (n = 15). (B) To analyze for anti‐metastatic potential, genomic DNA was extracted from the CAM and analyzed by qPCR with specific primers for human Alu sequences. Shown are means ± standard deviation (n = 8)

To evaluate the anti‐neoplastic activity of melflufen in vivo, we xenografted D492HER2 and MDA‐MB‐231 cells on chicken chorioallantoic membranes (CAM) and treated formed tumors with 50 µmol L^‐1^ melflufen or doxorubicin. Analysis of the tumors recovered after the treatment showed that both drugs were equally effective in inhibiting tumor growth compared to untreated control (Figure 5A). Interestingly, detection of MDA‐MB‐231 cells at the lower CAM by qPCR allowed for quantification of disseminated metastatic cells and revealed that melflufen is superior to doxorubicin in reducing the metastatic spread. While doxorubicin treatment resulted in around 60% metastatic regression, melflufen treatment led to a 90% regression rate (Figure 5B). As D492HER2 cells did not show any metastasis potential in this assay the effect could not be measured.

## 4 DISCUSSION

In this study, we have demonstrated that melflufen is an efficient drug in the treatment of breast cancer cells in vitro and in vivo. Furthermore, we showed that tumorigenic breast cancer cell lines MDA‐MB231 and D492HER2 are more sensitive to melflufen than the non‐tumorigenic D492 cell line. Importantly, we also showed that context matters, as cells cultured in 3D conditions were less sensitive to melflufen than cells in 2D monolayer culture. This is in agreement with previous studies that have shown that cellular behavior and drug sensitivity is different between 2D and 3D conditions.^8^, ^9^, ^10^, ^11^ Cells cultured in 2D have lost their polarity and their differentiation profile is different from the one seen in vivo resulting in altered cell signaling and gene expression, which eventually may affect drug sensitivity.^12^, ^13^ In contrast, cells cultured in 3D microenvironment in vitro can capture phenotypes as evidenced by the generation of structures akin to the condition in tissues and organs in the human body.^13^ Although 3D cultures are not perfect models, they represent a link between conventional cell cultures and in vivo conditions. Lovitt and co‐workers demonstrated recently that breast cancer cells acquired increased resistance against doxorubicin when cells were cultured in 3D, and this was due to altered β1‐integrin signaling.^8^ By blocking β1‐integrin signaling in MDA‐MB231 cells in 3D culture, they showed that the cells acquired an increased sensitivity toward doxorubicin treatment, which further supports accumulating evidence that the microenvironment is important for drug efficacy.^8^ For this reason, it is plausible that activation of survival pathways achieved in the 3D environent is contributing to decreased melflufen sensitivity as well.

Aminopeptidases, in particular CD13 also known as ANPEP, have been shown to be important activators of melflufen by cleavage of melflufen into the hydrophilic melphalan and *para*‐fluoro‐L‐phenylalanine.^14^, ^15^ In addition to its enzymatic activity, CD13 is also known for other functions such as receptor and signaling mechanisms.^16^ Due to high expression of aminopeptidases in many cancer types, aminopeptidase inhibitors are considered attractive tools for combination regimens in anti‐cancer treatment..^16^, ^17^ For breast cancer, it has been shown that aminopeptidase activity is increased in neoplastic tissue ^18^ and in a previous study 36.2% of studied breast cancer patients were positive to CD13 which significantly correlated with tumor type, neoangiogenesis and life expectancy.^19^

We demonstrated that melflufen activity was greatly reduced in cancer cells if cells were pretreated with the aminopeptidase inhibitor, bestatin. Furthermore, by enriching D492HER2 cells into CD13^high^ and CD13^low^ cell populations we showed that melflufen was significantly more active in the CD13^high^ population. Interestingly, we did not observe significant differences between CD13^high^ and CD13^low^ cell population in the nontumorigenic cell line D492. Analysis of endogenous CD13 expression levels revealed that D492HER2 cells have approximately an 1.6‐fold increase in CD13 expression compared to D492 cells (Figure S2). It is possible that the nontumorigenic D492 cell line is less dependent on CD13 expression and activity, while the presence of CD13 plays a more important role in the tumorigenic D492HER2 line. It could also be that CD13 and other aminopeptidases are used as a defense mechanism by tumorigenic cells whereas normal cells are less dependent on this function. For example, Dixon and co‐authors detected CD13 expression in breast epithelium and in 20% of breast cancer samples analyzed which affected doxorubicin resistance.^20^ Hence, slightly higher expression levels of CD13 could lead to higher enzyme activity in these cells and influence melflufen activity to a higher extent. Therefore, if CD13 activity is elevated and mediating resistance against doxorubicin melflufen application might be a good alternative treatment.

In that respect, it is of interest to note that to date, melflufen has been tested mostly in late stage or relapsed myeloma where cancer cells have survived several anti‐cancer treatments and therefore have probably intensified their survival mechanisms. As aminopeptidases are part of these survival mechanisms, this could explain why cancer cells are more sensitive to melflufen treatment than normal cells. Further studies as well as gene expression and protein activity analysis are needed to shed more light on this.

We also demonstrated that knock down of CD13 in D492HER2 cells reduced the efficacy of melflufen further demonstrating the importance of CD13 for the activity of melflufen in D492HER2 cells. The efficient activity of melflufen in cancer cells has been documented in several articles (reviewed in ^21^). Chauhan et al demonstrated that melflufen was highly efficient in killing myeloma cells, even cells that had acquired resistance to melphalan.^22^ Most studies on the efficacy of melflufen so far have been on haematological cancer, however, accumulating evidence points toward efficacy in solid cancer as well.^23^ In their paper, Delforoush and co‐workers demonstrated the efficacy of melflufen in lymphoma cells in vitro and in a xenograft model in vivo.^23^ Furthermore, Charlier et al demonstrated potency of melflufen on ovarian cancer cells in vitro and in vivo.^24^ Although CD13 has been in the spotlight as the main activator of melflufen, other aminopeptidases may also play a role. Fang and colleagues just recently showed that LAP3 is involved in migration and invasion of breast cancer cells.^25^ Accordingly, we demonstrated that knock down of LAP3 and DPP7 in D492HER2 cells reduced the positive activity of melflufen indicating that other aminopeptidases could play a role in breast cancer treatment, an important finding which needs further evaluation.When compared to the commonly used breast cancer drug doxorubicin we were able to show that melflufen is more potent both in vitro and in vivo. D492, D492HER2, and MDA‐MB231 cell lines were more sensitive to melflufen than doxorubicin, both in monolayer and in 3D culture. Furthermore, in an in vivo CAM assay, melflufen was similarly effective in decreasing tumor size stemming from D492HER2 and MDA‐MB231 cells, but was more effective in reducing the metastatic potential of MDA‐MB231 cells. The slightly higher efficacy of doxorubicin on tumor growth in the CAM assay is statistically not significant but could be explained by differences in bioavailability of the drugs due to rapid melflufen absorption.

Melflufen‐derived melphalan metabolites are known inducers of DNA damage by DNA double strand breaks.^26^ The phosphorylation of histone H2AX (ƴH2AX) at sites near DNA breaks is an early event in the response of mammalian cells to damage.^27^ In our assays, we could show robust phosphorylation of H2AX after melflufen application in a dose dependent manner. While statistical analysis of three independent experiments in both assays showed no or only minor significance between treated samples the results still represent a trend that phosphorylation of H2Ax is caused by treatment of cells with melflufen. In addition, Western blot analysis demonstrated that melflufen‐induced DNA damage resulted in apoptosis through activation of caspases‐3 and PARP cleavage. Previous studies have shown that melflufen induced DNA damage and apoptosis is not dependent on p53.^22^ This is of particular interest as the D492 and D492HER2 cells are p53 deficient ^2^ and therefore cannot undergo apoptosis through the regular p53 pathway. Our findings could have important clinical implications as it has been shown that p53 mutations occur in more aggressive forms of breast cancer ^28^ and are associated with worse prognosis.^29^ Therapeutic treatment with melflufen could present a valid therapeutic approach for these patient populations.

In recent years, organoid cultures have entered the spotlight as a powerful method for drug screening. It has been shown that culturing breast organoids in 3D retains the molecular signature of the original tumor.^30^ Having patient‐derived breast organoids profiled for expression of distinct type of aminopeptidases as well as p53 could identify those patients that could possibly benefit from melflufen treatment.

The fact that the highly lipophilic melflufen is converted to hydrophilic metabolites inside cells with high expression of aminopeptidases defines melflufen as a potential drug for targeted therapy and personalized medicine. However, due to its rapid intracellular uptake^21^ melflufens distribution and its ability to penetrate the core of solid tumors requires further investigation. Furthermore, it is not known how the microenviroment of the tumor influences the activity of melflufen as has been shown with doxorubicin.^8^ Therefore, it would be of the utmost interest to analyse the effects of melflufen in more detail in 3D culture and cell co‐culture.

## 5 CONCLUSION

In summary, we have demonstrated that the lipophilic peptide‐conjugated alkylator melflufen shows high efficacy in carcinogenic breast cell lines compared to their normal counterparts in vitro. This efficacy depends on aminopeptidases such as CD13, LAP3, and DPP7, which are shown to be associated with an aggressive phenotype. For this reason, melflufen might provide a new treatment option for breast cancer patients and deserves further investigation.

## 6 ETHICAL APPROVAL STATEMENT

No ethical approval is necessary.

## CONFLICT OF INTEREST

Fredrik Lehmann, Ana Slipicevic, and Nina Nupponen, declare employment as well as share options by Oncopeptides. Fredrik Lehman declares also ownerships of Oncopeptides shares. All remaining authors declared no conflict of interest.

## AUTHOR CONTRIBUTIONS

Conception and design: A. Schepsky., GA Traustadottir, A. Slipicevic., N. Nupponen., F. Lehmann., and T. Gudjonsson. Experimental design and data acquisition: A. Schepsky, GA Traustadottir, JP Joelsson, S. Ingthorsson, J. Knicker, JT Bergthorsson, A. Asbjarnarson, and Th. Gudjonsson. Analysis and interpretation of data: A. Schepsky and GA Traustadottir. Administrative, technical or material support: A. Slipicevic. Writing, review, and/or revision: A. Schepsky., GA Traustadottir, A. Slipicevic, F. Lehmann, and T. Gudjonsson. Study supervision: A. Slipicevic, F. Lehmann, and T. Gudjonsson.

## Supporting information


**Figure S1.**

**Figure S2.**

**Figure S3.**

**Figure S4.**

**Figure S5.**

## Data Availability

The data that support the findings of this study are available from the corresponding author upon reasonable request.
