# Supplementary figures and images for: Melflufen, a peptide‐conjugated alkylator, is an efficient anti‐neo‐plastic drug in breast cancer cell lines
Source: Cancer Med. 2020 Jul 27;9(18):6726–38. doi: 10.1002/cam4.3300 (PMC7520280; doi:10.1002/cam4.3300)

# Supplemental Figure 1

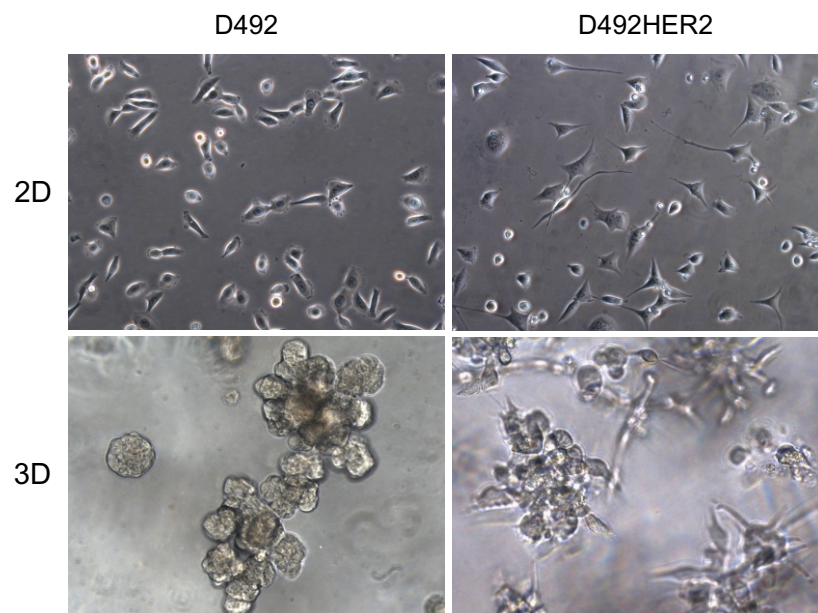

Supplement: Supplementary file 1 — Figure S1. [file CAM4-9-6726-s001.pdf]

# Supplemental Figure 2

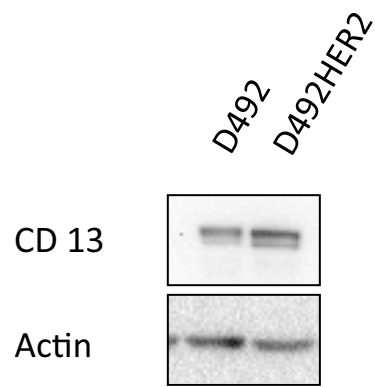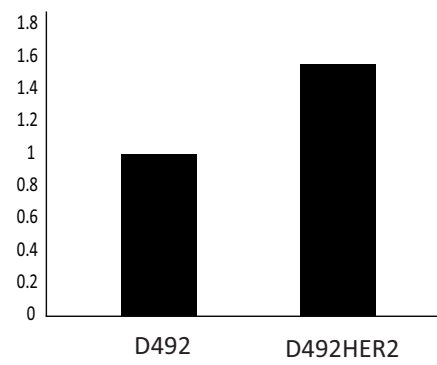

Supplement: Supplementary file 2 — Figure S2. [file CAM4-9-6726-s002.pdf]

### Supplemental Figure 3

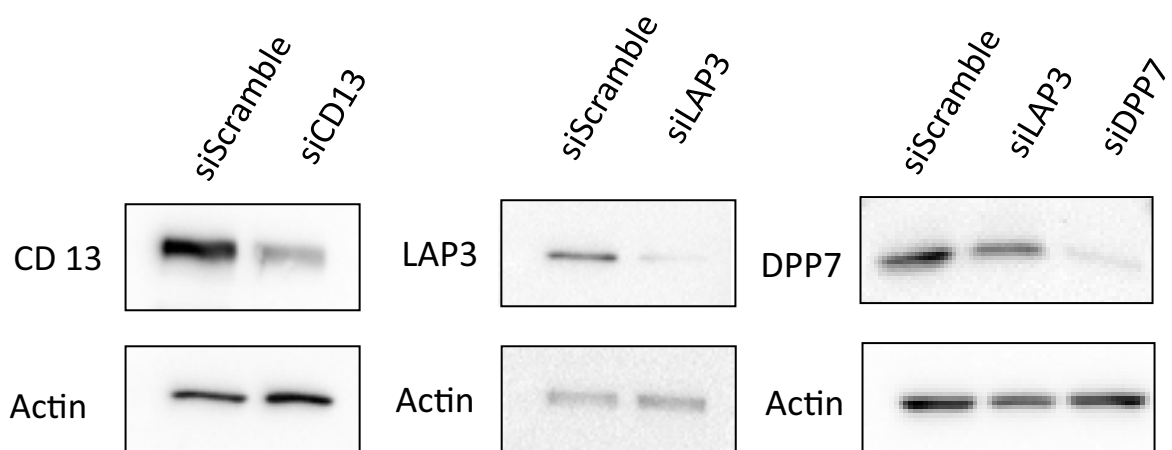

Supplement: Supplementary file 3 — Figure S3. [file CAM4-9-6726-s003.pdf]

Supplemental Figure 4

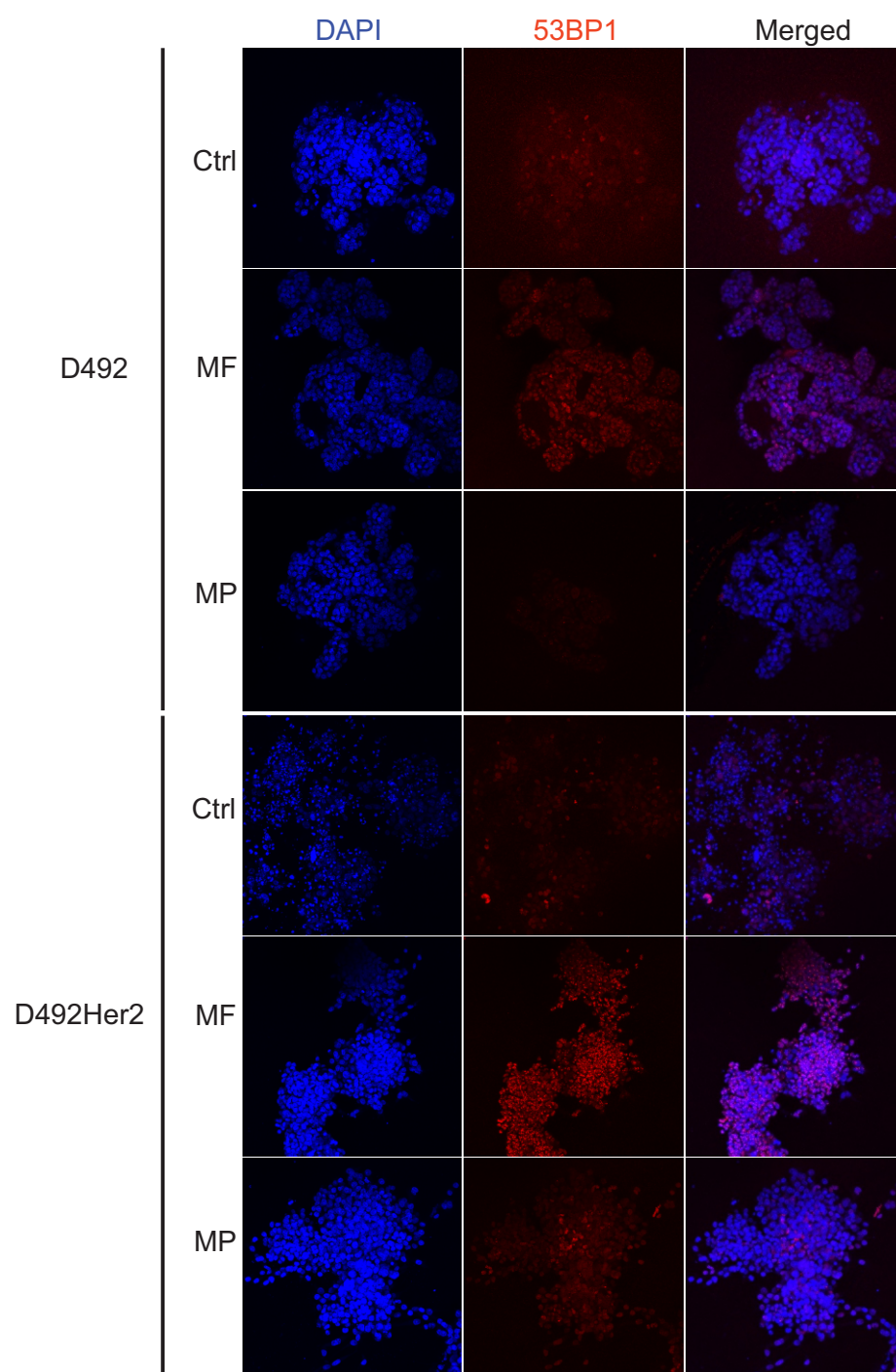

Supplement: Supplementary file 4 — Figure S4. [file CAM4-9-6726-s004.pdf]

# Supplemental Figure 5

**A**

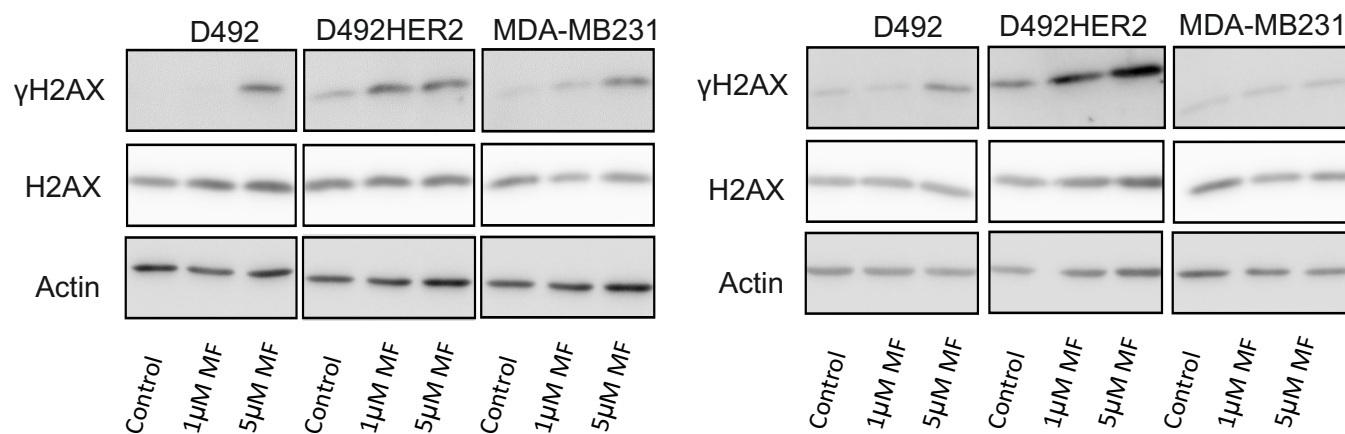

**B**

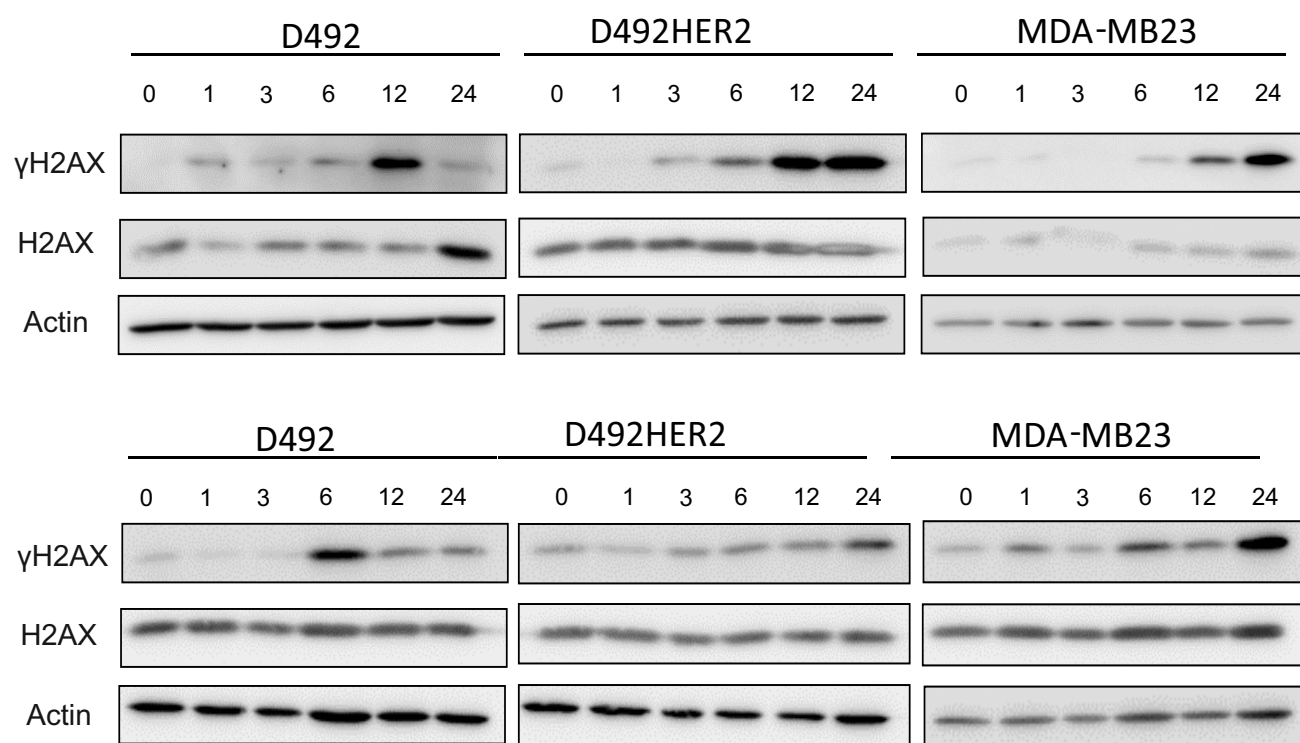

**C**

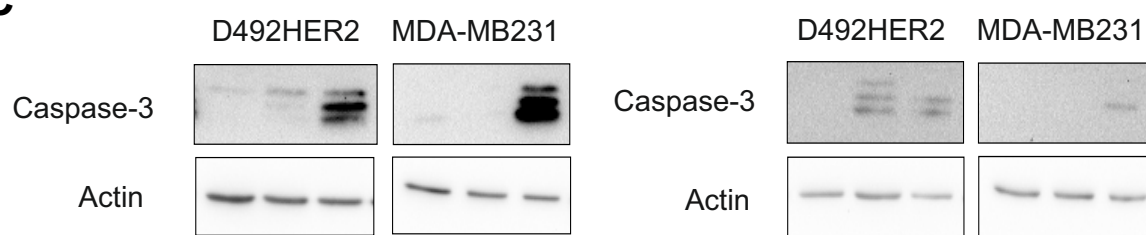

**D**

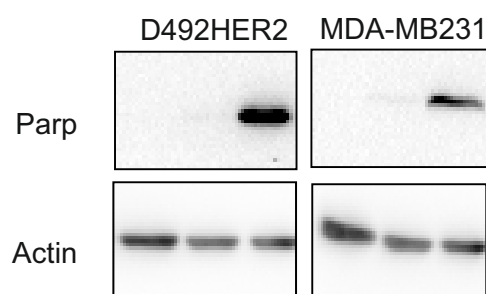

Supplement: Supplementary file 5 — Figure S5. [file CAM4-9-6726-s005.pdf]
